# Association of Cerebral Ischemia With Corneal Nerve Loss and Brain Atrophy in MCI and Dementia

**DOI:** 10.3389/fnins.2021.690896

**Published:** 2021-06-21

**Authors:** Georgios Ponirakis, Ahmed Elsotouhy, Hanadi Al Hamad, Surjith Vattoth, Ioannis N. Petropoulos, Adnan Khan, Hoda Gad, Fatima Al-Khayat, Mani Chandran, Marwan Ramadan, Marwa Elorrabi, Masharig Gadelseed, Rhia Tosino, Priya V. Gawhale, Maryam Alobaidi, Shafi Khan, Pravija Manikoth, Yasmin H. M. Abdelrahim, Noushad Thodi, Hamad Almuhannadi, Salma Al-Mohannadi, Fatema AlMarri, Murtaza Qazi, Ahmed Own, Ziyad R. Mahfoud, Ashfaq Shuaib, Rayaz A. Malik

**Affiliations:** ^1^Department of Medicine, Weill Cornell Medicine-Qatar, Qatar Foundation, Doha, Qatar; ^2^Faculty of Science and Engineering, Manchester Metropolitan University, Manchester, United Kingdom; ^3^Neuroradiology, Hamad General Hospital, Hamad Medical Corporation, Doha, Qatar; ^4^Geriatric and Memory Clinic, Rumailah Hospital, Hamad Medical Corporation, Doha, Qatar; ^5^Department of Radiology, University of Arkansas for Medical Sciences, Little Rock, AR, United States; ^6^Magnetic Resonance Imaging Unit, Rumailah Hospital, Hamad Medical Corporation, Doha, Qatar; ^7^Department of Medicine, University of Alberta, Edmonton, AB, Canada; ^8^Institute of Cardiovascular Science, University of Manchester, Manchester, United Kingdom

**Keywords:** ischemic lesions, surrogate marker, corneal confocal microscopy, corneal nerve fibers, brain atrophy, dementia, mild cognitive impairment

## Abstract

**Introduction:**

This study assessed the association of cerebral ischemia with neurodegeneration in mild cognitive impairment (MCI) and dementia.

**Methods:**

Subjects with MCI, dementia and controls underwent assessment of cognitive function, severity of brain ischemia, MRI brain volumetry and corneal confocal microscopy.

**Results:**

Of 63 subjects with MCI (*n* = 44) and dementia (*n* = 19), 11 had no ischemia, 32 had subcortical ischemia and 20 had both subcortical and cortical ischemia. Brain volume and corneal nerve measures were comparable between subjects with subcortical ischemia and no ischemia. However, subjects with subcortical and cortical ischemia had a lower hippocampal volume (*P* < 0.01), corneal nerve fiber length (*P* < 0.05) and larger ventricular volume (*P* < 0.05) compared to those with subcortical ischemia and lower corneal nerve fiber density (*P* < 0.05) compared to those without ischemia.

**Discussion:**

Cerebral ischemia was associated with cognitive impairment, brain atrophy and corneal nerve loss in MCI and dementia.

## Introduction

Cerebrovascular ischemic lesions are present in 11–94% of people aged ≥60 years (Debette and Markus, 2010). They increase the risk of cognitive impairment (Mungas et al., 2001) and dementia (Prins et al., 2004; Debette et al., 2010), depending on the person’s age, education and lifestyle, as well as the location and size of ischemic load (Roh and Lee, 2014). Ischemic lesions are incomplete infarctions due to reduced cerebral blood flow caused by critical stenosis in small vessels in the white matter of the brain (Roh and Lee, 2014) and can increase and decrease in size (Wardlaw et al., 2015). Management of hypertension (Dufouil et al., 2001; de Leeuw et al., 2002) and diabetes (McNay, 2005; Geijselaers et al., 2017) and antiplatelet therapy (Gorelick et al., 2011), particularly calcium-channel blockers (Pantoni et al., 2000) may prevent or reduce ischemic lesions.

There is limited evidence on the relationship between the presence and severity of ischemic lesions and neurodegeneration (Nitkunan et al., 2011; Kamran et al., 2020). Patients with ischemic lesions and lacunar stroke have impaired executive function and reduced brain volume (Nitkunan et al., 2011). Corneal confocal microscopy (CCM) has shown significant corneal nerve loss in patients with TIA (Gad et al., 2019) and acute ischemic stroke (Khan et al., 2018) and has been associated with the presence of white matter hyperintensities after adjusting for age, diabetes, dyslipidemia and smoking (Kamran et al., 2020). We have also previously reported that corneal nerve loss is associated with the severity of cognitive impairment and physical disability in patients with mild cognitive impairment (MCI) and dementia (Ponirakis et al., 2019a; Al-Janahi et al., 2020).

The aim of this study was to assess whether the presence and severity of cerebral ischemic lesions was associated with neurodegeneration quantified by MRI brain volumetry and CCM in patients with MCI and dementia. This study excluded patients with diabetes as this is a confounding factor for corneal nerve loss (Tavakoli et al., 2010).

## Materials and Methods

Patients with MCI and dementia, including Alzheimer’s disease (AD), vascular dementia (VaD) and mixed dementia, and healthy controls aged 50–85 years old were recruited from the Geriatric and Memory clinic in Rumailah Hospital, Doha, Qatar between 18/09/16 and 31/07/19. Patients with severe anxiety, severe depression, Parkinson’s disease, frontotemporal dementia and Lewy body dementia, hypomania, and severe dementia who were unable to cooperate were excluded. Additionally, patients with peripheral neuropathy including diabetes, vitamin B_12_ deficiency, hypothyroidism, HIV infection and hepatitis C were excluded. Patients with dry eyes, corneal dystrophies, ocular trauma or surgery in the preceding 12 months were excluded. This study was approved by the Institutional Review Board of Weill Cornell Medicine in Qatar (15-00019) and Hamad Medical Corporation (RP14494/14) and all participants gave informed consent to take part in the study. The research adhered to the tenets of the declaration of Helsinki.

### Demographic and Metabolic Measures

Age, gender, ethnicity, blood pressure, weight, body mass index (BMI), HbA1c, cholesterol, triglycerides, thyroid stimulating hormone (TSH), free thyroxine (FT4), and vitamin B_12_ were recorded.

### Cognitive Function Assessment

Cognitive function was assessed using the Montreal Cognitive Assessment (MoCA) test. The MoCA assesses seven cognitive domains including visuospatial/executive, naming, memory, attention, language, abstraction and delayed recall giving a total score of 30. A score of ≤26 indicates cognitive impairment. A point was added for individuals who had formal education ≤6th grade. Cognitive symptom duration was estimated from the clinical history obtained from relatives and participants.

### Diagnosis

The diagnosis of MCI and dementia, including AD, VaD and mixed AD were based on the ICD-10 criteria (World Health Organization, 1992). The diagnosis was made according to consensus decision by geriatricians, geriatric psychiatrists and neurologists to exclude reversible, complex and young-onset dementia. The diagnoses of MCI and dementia were based on a patient history and examination, which include (1) presenting complaint and history of illness; (2) comprehensive history of each of the cognitive domains using MoCA; (3) psychiatric history for ruling out depression, mood disorders, and psychosis; (4) medical history including episodes of delirium and other medical comorbidities; (5) medication history; (6) functional history of basic daily living activities. A comprehensive organic work-up including blood tests and brain imaging was undertaken to exclude other potentially reversible causes of cognitive decline such as tumors, subdural hematoma or normal pressure hydrocephalus. The diagnosis of AD was based on typical features of AD on MRI, including volume loss of hippocampi, entorhinal cortex, and amygdala. The diagnosis of mixed AD was based on the presence of AD and significant vascular changes. The diagnosis of probable or possible VaD was based on multiple large vessel infarcts or a single strategically placed infarct in the angular gyrus, thalamus, basal forebrain, or posterior (PCA) or anterior cerebral artery (ACA) territories, and multiple lacunes in basal ganglia and white matter, extensive periventricular white matter lesions or combinations thereof.

### Brain MRI Acquisition

MRI was performed on a superconductive magnet operated at 3T (Skyra, Siemens) at the MRI unit in Rumailah Hospital. The subject’s head was immobilized with a head holder to minimize motion artifacts. A T1-weighted 3D magnetisation prepared rapid acquisition gradient echo sequence (MPRAGE) was obtained in the sagittal plane with a 1 mm slice thickness, repetition time of 1,900 ms, echo time of 2.67 and 2.46 ms, inversion time of 1,100 and 900 ms, flip angle of 9 degree and 15 degree, and FOV = 240 × 100. Coronal and axial reformatted MPRAGE images are reconstructed from the sagittal 3D sequence.

### Ischemic Lesion Assessment

The presence of ischemic lesions was defined as hyperintense foci on T2 and FLAIR. Small vessel disease (SVD) was assessed by the presence of white matter hyperintensities (WMH) in cortical, subcortical or both regions. Infarcts including lacunes, large infarcts and hemorrhage were not included in the analysis. Foci that were hyperintense on T2 and showed central low signal with a peripheral rim of hyperintensity on FLAIR were defined as lacunes. Larger areas of gliosis/encephalomalacia following a vascular pattern or diffusion restricting acute ischemic lesions were defined as infarcts.

Subcortical ischemia was based on the presence of ischemic lesions located in the subcortical white matter, deep gray nuclei including basal ganglia, thalami, and mesial temporal lobe. Cortical ischemia was based on the presence of ischemic lesions located in the cerebral convexity cortex.

### Brain Volume Analysis

MRI T1-weighted 3D MPRAGE sequences were processed using NeuroQuant (NQ), an FDA approved fully automated software (Brewer et al., 2009; Stelmokas et al., 2017) to measure brain volumes. The brain volume was adjusted for percentage of intracranial volume (ICV) which includes all segmented structures to minimize the impact of the head size as a confounding factor. The ICV percentage of the whole brain, cortical gray matter, ventricle, hippocampi, frontal, temporal and parietal lobe are included in this study.

### Corneal Confocal Microscopy

CCM analysis was performed with the Heidelberg Retinal Tomograph III Rostock Cornea Module (Heidelberg Engineering GmbH, Heidelberg, Germany). The cornea was locally anesthetized by instilling 1 drop of 0.4% benoxinate hydrochloride (Chauvin Pharmaceuticals, Chefaro, United Kingdom) and Viscotears gel (Carbomer 980, 0.2%, Novartis, United Kingdom) was used as the coupling agent between the cornea and the TomoCap as well as between the TomoCap and the objective lens. Subjects were instructed to fixate on a target with the eye not being examined. Several scans of the sub-basal nerve plexus in the central cornea were captured per eye for ∼2 min. The field of view of each image is 400 × 400 μm. At a separate time, three high clarity images per eye were selected by one researcher blind to the patient diagnosis using established criteria based on depth, focus position and contrast (Kalteniece et al., 2017). Corneal nerve fiber density (CNFD) (fibers/mm^2^), branch density (CNBD) (branches/mm^2^) and fiber length (CNFL) (total fiber length mm/mm^2^) were quantified manually using CCMetrics, a validated image analysis software (Dabbah et al., 2011).

### Peripheral Neuropathy Assessments

Vibration perception threshold (VPT) as a measure of large myelinated nerve fiber function was assessed using a Neurothesiometer (Horwell Scientific Laboratory Supplies) on the pulp of the large toe on both feet and the average value of three measurements was recorded as a VPT in Volts (V) ranging from 0 to 50 V.

Sudomotor function was assessed by measuring electrochemical skin conductance (ESC) in microSiemens (μS) using Sudoscan (Impeto Medical SAS). It evaluates sympathetic innervation based on sweat chloride concentrations generated by the sweat gland in response to the voltage applied (Casellini et al., 2013).

### Group Definition

This study assessed four groups: (1) healthy control subjects without cognitive impairment or dementia; (2) subjects with MCI or dementia without cerebral ischemia; (3) subjects with MCI or dementia with subcortical ischemia, and (4) subjects with MCI or dementia with cortical and subcortical ischemia.

### Statistical Analysis

Given that the difference in neuronal injury measured by MRI brain volumetry and CCM between subjects without and with cortical and subcortical ischemic lesions have not been studied before, the results were analyzed as an exploratory study and not adjusted for multiple testing or multiple comparisons (Rothman, 1990).

Variables were compared between controls, subjects without ischemic lesions, with subcortical ischemia and both subcortical and cortical ischemia using one-way analysis of variance (ANOVA) with least significant difference (LSD) *post hoc* test for pairwise comparisons and categorical outcomes were compared using Chi-square test. The age-adjusted mean difference in the volume of different brain structures and corneal nerve measures between the three groups, excluding controls were estimated using covariance (ANCOVA) with LSD test for *post hoc* comparisons. Variables were summarized using means and standard deviations for numeric variables and frequency distribution for categorical variables.

All analyses were performed using IBM-SPSS (version 26; SPSS Inc., Armonk, NY). A two-tailed *P*-value of ≤0.05 was considered significant.

## Results

### Demographic and Clinical Characteristics

Of the 90 subjects studied, 27 were controls, 44 had MCI, and 19 had dementia. The clinical characteristics of controls, those without ischemia (*n* = 11), subcortical ischemia (*n* = 32) and both cortical and subcortical ischemia (*n* = 20) are summarized in Table 1. Gender (*P* = 0.16), and the percentage of subjects with hypertension (*P* = 0.71), MCI and dementia (*P* = 0.25) were comparable between the groups. Controls and subjects without ischemia were significantly younger compared to those with subcortical ischemia (*P* < 0.05) and both cortical and subcortical ischemia (*P* < 0.01), but age was comparable between the latter two groups (*P* = 0.30). The duration of cognitive impairment was comparable between those without ischemia, subcortical ischemia and both cortical and subcortical ischemia. Systolic (SBP), diastolic blood pressure (DBP), HbA1c, total cholesterol, and triglycerides were comparable between the groups. Body weight and BMI were lower in subjects with both cortical and subcortical ischemia compared to subjects with subcortical ischemia (*P* < 0.05).

**TABLE 1:** Comparison of demographic and clinical characteristics between healthy controls and no ischemia, subcortical ischemia and both cortical and subcortical ischemia in subjects with MCI and dementia.

	Healthy controls (*n* = 27)	No ischemia (*n* = 11)	Subcortical ischemia (*n* = 32)	Cortical and subcortical ischemia (*n* = 20)	*P*-value^*a*^	*P-*value^*b*^	*P-*value^*c*^
Mild cognitive impairment, *n* (%)	N/A	10 (22.7)	21 (47.7)	13 (29.5)	*P*-value = 0.25		
Dementia	N/A	1 (5.3)	11 (57.9)	7 (36.8)			
Age, years	63.2 ± 8.9	63.0 ± 7.3	70.0 ± 8.8^†^	72.5 ± 7.5^††^	<0.05	<0.01	0.31
Duration of cognitive impairment	N/A	3.9 ± 5.9	2.0 ± 1.9	2.6 ± 2.3	0.11	0.27	0.57
Female, *n* (%)	7 (25.9)	7 (63.6)	12 (37.5)	6 (30.0)	*P*-value = 0.16		
Hypertension, *n* (%)	14 (58.3)	7 (63.6)	16 (50.0)	13 (65.0)	*P*-value = 0.71		
Systolic BP, mmHg	138.0 ± 13.9	137.9 ± 18	135.8 ± 13.3	141.1 ± 24.8	0.66	0.63	0.25
Diastolic BP, mmHg	76.0 ± 10.6	76.7 ± 8.6	73.3 ± 7.1	73.9 ± 8.4	0.28	0.31	0.99
Weight, kg	74.8 ± 8.8	74.1 ± 19.3	82.4 ± 16.1	72.6 ± 12.3	0.13	0.77	<0.05
BMI, kg/m^2^	28.0 ± 3.4	28.7 ± 7.4	31.8 ± 6.7^†^	27.3 ± 4.9	0.12	0.51	<0.01
HbA1c, %	5.7 ± 0.4	5.7 ± 0.5	5.6 ± 0.6	5.6 ± 0.3	0.82	0.83	0.99
Total cholesterol, mmol/L	5.2 ± 0.9	4.8 ± 0.6	4.9 ± 1.2	5.2 ± 0.7	0.89	0.37	0.33
Triglycerides, mmol/L	1.4 ± 0.6	1.1 ± 0.6	1.4 ± 0.7	1.5 ± 0.9	0.33	0.19	0.58

*^a^No ischemia vs. subcortical ischemia. ^b^No ischemia vs. cortical and subcortical ischemia. ^c^Subcortical ischemia vs. cortical and subcortical ischemia. Data from 90 subjects presented as mean ± standard deviation for numeric variables and frequency distribution for categorical variables. Continuous and categorical variables were compared using one-way ANOVA with LSD test for post hoc comparisons and Chi-square test, respectively. Differences between control subjects and subjects with MCI/dementia are indicated as ^‡^P ≤ 0.05, ^†^P ≤ 0.01, ^††^P ≤ 0.001, P < 0.0001.*

### Cognitive Function (Table 2)

Based on the MoCA, global cognitive function was significantly lower in subjects with both cortical and subcortical ischemia (*P* < 0.01), but not in those with subcortical ischemia when compared to those without ischemia (*P* = 0.28) (Table 2). Compared to those without ischemia a lower percentage of subjects with subcortical ischemia and both cortical and subcortical ischemia successfully completed the tests for visuospatial executive function (22.2% and 11.8% vs. 63.6%, *P* < 0.05) and orientation (44.4% and 29.4% vs. 81.8%, *P* = 0.01). The percentage of subjects with subcortical ischemia and both cortical and subcortical ischemia who successfully completed the attention test was non-significantly lower compared to those without ischemia (37.0% and 35.3% vs. 72.7%, *P* = 0.63). Performance in the other domains, including naming, sentence repetition and letter fluency, abstraction to connect related concepts and delayed recall memory test were comparable between all three groups.

**TABLE 2:** Comparison of cognitive function, corneal nerve fiber morphology and volumetric brain MRI between healthy controls and no ischemia, subcortical ischemia and both cortical and subcortical ischemia in subjects with MCI and dementia.

	Healthy controls (*n* = 27)	No ischemia (*n* = 11)	Subcortical ischemia (*n* = 32)	Cortical and subcortical ischemia (*n* = 20)	*P*-value^*a*^	*P-*value^*b*^	*P-*value^*c*^
MoCA	27.4 ± 3.9	24.3 ± 5.7	20.8 ± 6.0^†††^	18.8 ± 6.1^†††^	0.10	<0.01	0.28
CNFD, fibers/mm^2^	32.9 ± 6.3	31.2 ± 6.7	27.0 ± 9.0^†^	21.7 ± 9.1^†††^	0.18	<0.01	<0.05
CNBD, branches/mm^2^	105.2 ± 51.1	78.3 ± 33.5	69.6 ± 45.4^†^	46.4 ± 28.5^†††^	0.57	0.05	0.06
CNFL, mm/mm^2^	24.5 ± 5.6	21.2 ± 5.1	19.3 ± 6.9^†^	14.9 ± 5.8^†††^	0.38	<0.01	0.01
CNBD:CNFD ratio	3.2 ± 1.5	2.5 ± 0.9	2.5 ± 1.2^‡^	2.2 ± 1.4^†^	1.00	0.53	0.42
VPT, Volts	9.5 ± 6.0	16.5 ± 13.4‡	18.3 ± 8.7^††^	18.6 ± 10.4^††^	0.61	0.57	0.92
ESC feet, μS	63.6 ± 19.5	62.4 ± 18.9	58.7 ± 18.5	54.6 ± 20.4	0.65	0.36	0.48
Cortical gray matter, ICV%	29.1 ± 2.7	28.9 ± 3.5	28.2 ± 3.8	27.3 ± 4.1	0.61	0.26	0.39
Hippocampus, ICV%	0.50 ± 0.05	0.47 ± 0.08	0.43 ± 0.08^††^	0.35 ± 0.07^†††^	0.20	<0.0001	<0.01
Ventricle, ICV%	2.4 ± 1.3	2.6 ± 2.0	3.0 ± 1.9	4.4 ± 1.3^††^	0.56	0.01	<0.01
Frontal lobe, ICV%	10.6 ± 0.9	10.6 ± 1.1	10.2 ± 1.6	9.6 ± 1.5^‡^	0.46	0.08	0.16
Temporal lobe, ICV%	7.6 ± 0.9	7.4 ± 1.1	7.4 ± 1.1	7.0 ± 1.2	1.00	0.31	0.18
Parietal lobe, ICV%	6.6 ± 0.8	6.8 ± 1.1	6.2 ± 0.9	6.1 ± 0.8	0.08	0.06	0.75
Whole brain, ICV%	74.0 ± 3.3	74.1 ± 3.1	71.0 ± 4.6^†^	69.2 ± 3.2^††^	<0.05	<0.01	0.13

*^a^No ischemia vs. subcortical ischemia. ^b^No ischemia vs. cortical and subcortical ischemia. ^c^Subcortical ischemia vs. cortical and subcortical ischemia. Variables from 90 subjects presented as mean ± standard deviation were compared using one-way ANOVA with LSD test for post hoc comparisons. Differences between control subjects and subjects with MCI/dementia are indicated as ^‡^P ≤ 0.05, ^†^P ≤ 0.01, ^††^P ≤ 0.001, ^†††^P ≤ 0.0001. MoCA, Montreal cognitive assessment; CNFD, corneal nerve fiber density; CNFL, length; CNBD, branch density; VPT, vibration perception threshold; ESC, electrochemical skin conductance; ICV, intra cranial volume.*

### Corneal Nerve Fiber Measures

**FIGURE 1:**
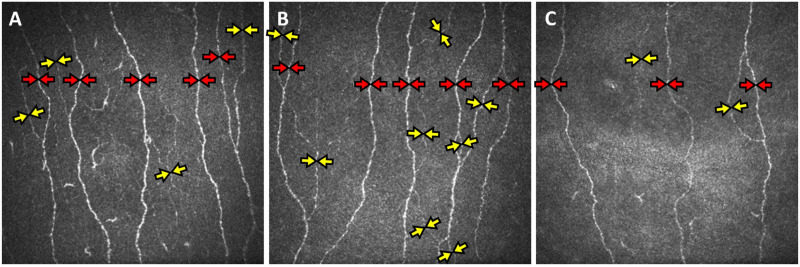
Corneal confocal microscopy (CCM) images of the sub-basal nerve plexus of subjects with mild cognitive impairment without cerebral ischemia **(A)**, with subcortical ischemia **(B)**, and cortical and subcortical ischemia **(C)** showing reduced main nerve fibers (red arrows), branches (yellow arrows) and total fiber length in subjects with cortical and subcortical ischemia.

Corneal nerve fiber measures were significantly lower in subjects with ischemia compared to healthy controls (*P* < 0.01); but were comparable between health controls and subjects with MCI/dementia without ischemia (Table 2 and Figure 1).

### Subcortical Ischemia vs. No Ischemia

There was no significant difference in CNFD, CNBD, CNFL and CNBD:CNFD ratio between patients with subcortical ischemia and no ischemia.

### Cortical and Subcortical Ischemia vs. No Ischemia

CNFD (*P* < 0.01), CNBD (*P* = 0.05) and CNFL (*P* < 0.01) were significantly lower in patients with cortical and subcortical ischemia compared to no ischemia.

### Cortical and Subcortical Ischemia vs. Subcortical Ischemia

CNFD (*P* < 0.05) and CNFL (*P* = 0.01) were significantly lower in subjects with cortical and subcortical ischemia compared to subcortical ischemia.

After adjusting for age, subjects with both cortical and subcortical ischemia had a significantly lower CNFD (−7.2 fibers/mm^2^, 95% CI −14.1 to 0.4, *P* < 0.05) compared to no ischemia and a significantly lower CNFL (−4.0 mm/mm^2^, 95% CI −7.5 to −0.5, *P* < 0.05) compared to those with subcortical ischemia.

There was no significant difference in the CNBD:CNFD ratio between the three groups.

### MRI Brain Volumetry

**FIGURE 2:**
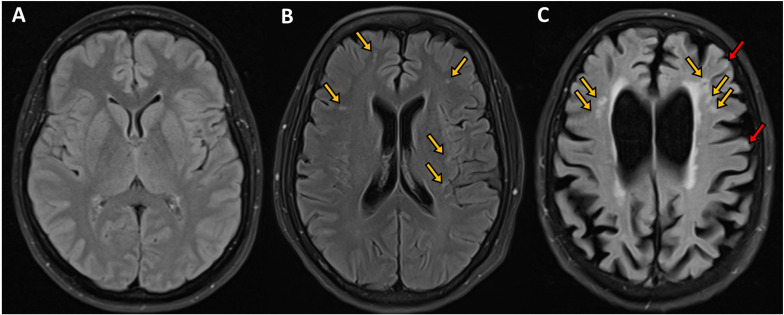
Axial FLAIR images of the brain of subjects with mild cognitive impairment showing **(A)** normal appearing visualized brain parenchyma with no evidence of ischemia, infarction or microbleeds, **(B)** bilateral scattered subcortical white matter ischemic changes in the form of hyperintense (bright foci) secondary to microangiopathy shown in orange arrows, and **(C)** moderate brain atrophy with cortical and subcortical ischemic foci in the form of bright signal intensity shown by orange and red arrows, respectively. Courtesy of Dr. Ahmed Elsotouhy.

The volume of hippocampus (*P* < 0.0001), whole brain (*P* < 0.001) and frontal lobe (*P* < 0.05) were significantly lower and ventricle volume was significantly higher (<0.001) in subjects with cortical and subcortical ischemia compared to healthy controls, but the volume of cortical gray matter, temporal lobe and parietal lobe were comparable (Table 2 and Figure 2). The volume of hippocampus (*P* < 0.001) and whole brain (*P* < 0.01) were significantly lower in subjects with subcortical ischemia compared to healthy controls. There was no difference in MRI brain volume between health controls and subjects with MCI/dementia without ischemia.

### Subcortical Ischemia vs. No Ischemia

Only whole brain volume (*P* < 0.05) was significantly lower in patients with subcortical ischemia compared to no ischemia.

### Cortical and Subcortical Ischemia vs. No Ischemia

Whole brain volume (*P* < 0.01) and hippocampal volume (*P* < 0.0001) were lower and ventricular volume (*P* = 0.01) was higher in patients with cortical and subcortical ischemia compared to no ischemia.

### Cortical and Subcortical Ischemia vs. Subcortical Ischemia

Hippocampal volume (*P* < 0.01) was lower and ventricular volume (*P* < 0.01) was higher in patients with cortical and subcortical ischemia compared to subcortical ischemia.

After adjusting for age, subjects with cortical and subcortical ischemia had a significantly lower hippocampal volume (−0.06 ICV%, 95% CI −0.10 to 0.02, *P* < 0.01) and larger ventricular volume (1.27 ICV%, 95% CI 0.25-2.29, *P* < 0.05) compared to those with subcortical ischemia. There was no difference in hippocampal volume (*P* = 0.08) and ventricular volume (*P* = 0.14) between subjects with subcortical ischemia and no ischemia.

There was no significant difference in cortical gray matter, frontal, temporal, and parietal lobe volumes between the three groups.

### Peripheral Neuropathy Assessments

Vibration perception threshold (VPT) and electrochemical skin conductance (ESC) on the feet, were comparable between those without ischemia, subcortical ischemia and cortical and subcortical ischemia, even after adjusting for age (*P* = 0.82). VPT was significantly higher in those with MCI and dementia compared to controls (*P* ≤ 0.05) (Table 2).

## Discussion

In this study, cortical and subcortical ischemia was associated with neurodegeneration quantified by MRI brain volumetry and corneal confocal microscopy (CCM) in patients with MCI and dementia. It was also associated with reduced global cognitive function, particularly executive function, and orientation.

Unlike vascular dementia (VaD) caused by multiple or strategic infarction or hemorrhage, which develops relatively quickly in patients with stroke, mixed dementia attributed to amyloid deposits and ischemic lesions develops relatively slowly (Pendlebury, 2009; Pendlebury and Rothwell, 2009). Ischemic lesions arise as a consequence of chronically reduced blood flow to the white matter due to critical stenosis of the cortical medullary branches (Roh and Lee, 2014) and are present in approximately 50% of patients with Alzheimer’s disease (AD) (Wang et al., 2012). Indeed, ischemic lesions can be present in 11–94% of people aged ≥60 years (Debette and Markus, 2010) and can increase in size, shrink or in rare instances, disappear (Wardlaw et al., 2015).

There is a need for reliable surrogate biomarkers of neurodegeneration to identify patients at a higher risk for cognitive impairment and dementia. MRI brain volumetry has been suggested as a surrogate marker of neurodegeneration (Nitkunan et al., 2011) especially as brain atrophy is associated with impairment in executive function in patients with ischemic lesions (Nitkunan et al., 2011). This study confirms that ischemic lesions are associated with impaired executive function and orientation in patients with MCI and dementia. Furthermore, this study shows that MRI brain volumetry showed significantly reduced hippocampal volume and increased ventricular volume in patients with both cortical and subcortical ischemia compared to those with subcortical ischemia. Ischemic lesions have been associated with an increase in the risk of cognitive impairment (Mungas et al., 2001) and dementia (Prins et al., 2004; Debette et al., 2010). This study now adds to this by showing that cortical and subcortical ischemia is an independent risk factor for neurodegeneration in patients with MCI and dementia (Nitkunan et al., 2011; Kamran et al., 2020).

We have previously shown that reduced corneal nerve fiber loss was associated with increased white matter hyperintensities in patients with acute ischemic stroke (Kamran et al., 2020). We have also demonstrated significant corneal nerve loss in patients with MCI and dementia, which was associated with the severity of cognitive impairment and disability (Ponirakis et al., 2019a). Moreover we have recently shown that the diagnostic accuracy of CCM was high and comparable with MRI based medial temporal lobe atrophy (MTA) rating for dementia and was superior in MCI (Al-Janahi et al., 2020). This study shows that patients with both cortical and subcortical ischemia, but not subcortical ischemia alone, have evidence of corneal nerve loss suggesting that more severe ischemia is associated with neurodegeneration in patients with MCI and dementia.

The prevalence of ischemic lesions increases with age (Smith et al., 2017), hypertension (Dufouil et al., 2001; de Leeuw et al., 2002) and diabetes (McNay, 2005; Geijselaers et al., 2017) and may improve with antiplatelet therapy (Gorelick et al., 2011) and management of hypertension (Dufouil et al., 2001; de Leeuw et al., 2002) and diabetes (McNay, 2005; Geijselaers et al., 2017). These same risk factors have been related to corneal nerve degeneration (Ponirakis et al., 2019b) and improvement in blood pressure, lipids and glycemic control is associated with corneal nerve regeneration (Tavakoli et al., 2011; Azmi et al., 2019).

A significant limitation of this study is the small sample size due to the need to exclude patients with diabetes, a confounding factor for corneal nerve loss. The lack of differences in MRI brain volumetry and corneal nerve fiber measures between control subjects and those without ischemia might be attributed to the fact that all subjects without ischemia had MCI apart from one patient who had dementia. The causal inference of ischemic lesions on brain atrophy and corneal nerve fiber loss cannot be ascertained because this is a cross-sectional study.

This study shows that cortical and subcortical ischemia was associated with impaired global cognitive function, brain atrophy and corneal nerve fiber loss in patients with MCI and dementia. CCM and MRI brain volumetry may act as non-invasive surrogate markers of neurodegeneration associated with cortical and subcortical ischemia in patients with MCI and dementia. Larger, longitudinal studies are needed to evaluate the utility of CCM as a surrogate marker of neurodegeneration taking into account cortical and subcortical ischemia in patients with MCI and dementia.

## Data Availability Statement

The raw data supporting the conclusions of this article will be made available by the authors, without undue reservation.

## Ethics Statement

The studies involving human participants were reviewed and approved by IRB Weill Cornell Medicine. The patients/participants provided their written informed consent to participate in this study.

## Author Contributions

RM and GP had full access to all the data in the study and take responsibility for the integrity of the data and the accuracy of the data analysis, study concept and design, and drafting of the manuscript. GP, ZM, and RM: statistical analysis. RM: obtained funding. All authors: acquisition, analysis, or interpretation of data, critical revision of the manuscript for important intellectual content, and administrative, technical, or material support. All authors have read and approved the final manuscript.

## Conflict of Interest

The authors declare that the research was conducted in the absence of any commercial or financial relationships that could be construed as a potential conflict of interest.
